# Alcohol and Cancer: Mechanisms and Therapies

**DOI:** 10.3390/biom7030061

**Published:** 2017-08-14

**Authors:** Anuradha Ratna, Pranoti Mandrekar

**Affiliations:** Department of Medicine, University of Massachusetts Medical School, Worcester, MA 01605, USA; anuradha.ratna@umassmed.edu

**Keywords:** alcohol, metabolism, cancer risk, animal models, immunosuppression, immunotherapy

## Abstract

Several scientific and clinical studies have shown an association between chronic alcohol consumption and the occurrence of cancer in humans. The mechanism for alcohol-induced carcinogenesis has not been fully understood, although plausible events include genotoxic effects of acetaldehyde, cytochrome P450 2E1 (CYP2E1)-mediated generation of reactive oxygen species, aberrant metabolism of folate and retinoids, increased estrogen, and genetic polymorphisms. Here, we summarize the impact of alcohol drinking on the risk of cancer development and potential underlying molecular mechanisms. The interactions between alcohol abuse, anti-tumor immune response, tumor growth, and metastasis are complex. However, multiple studies have linked the immunosuppressive effects of alcohol with tumor progression and metastasis. The influence of alcohol on the host immune system and the development of possible effective immunotherapy for cancer in alcoholics are also discussed here. The conclusive biological effects of alcohol on tumor progression and malignancy have not been investigated extensively using an animal model that mimics the human disease. This review provides insights into cancer pathogenesis in alcoholics, alcohol and immune interactions in different cancers, and scope and future of targeted immunotherapeutic modalities in patients with alcohol abuse.

## 1. Introduction

Chronic alcohol consumption is a major health concern worldwide, and may lead to damage of almost every organ of the body. According to the Centers for Disease Control and Prevention, nearly 88,000 people die from alcohol-related causes annually in the United States [1]. Globally, in 2012, 3.3 million deaths, or 5.9% of total deaths, were attributable to alcohol consumption [2]. In 2014, about 16.3 million adults (ages 18 and older) and an estimated 679,000 adolescents (ages 12–17) had an alcohol use disorder in the United States [3]. Based on previous studies, there is a strong scientific consensus of an association between drinking alcohol and several types of cancer, including those of liver, breast, upper aerodigestive tract (mouth, oropharynx, hypopharynx, and esophagus), pancreas and colon [4,5]. Whether alcohol impacts cancer initiation or progression is not well understood [6].

**Figure 1:**
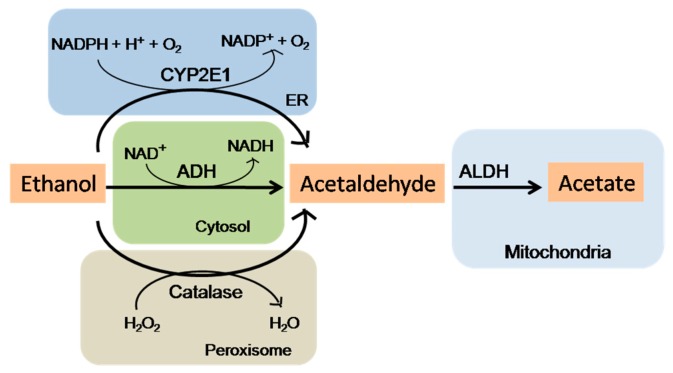
Pathways of ethanol metabolism. Ethanol is oxidized mainly by cytosolic alcohol dehydrogenase (ADH) to acetaldehyde. Acetaldehyde then enters the mitochondria where it is oxidized to acetate by mitochondrial aldehyde dehydrogenase (ALDH). Another major pathway of ethanol metabolism includes its oxidation in microsomes by cytochrome P450 2E1 (CYP2E1) enzyme and requires nicotinamide adenine dinucleotide phosphate (NADPH) instead of nicotinamide adenine dinucleotide (NAD^+^) as for ADH. Reactive oxygen species (ROS) are formed due to alcohol metabolism by CYP2E1 and the re-oxidation of NADH in the mitochondria. A catalase-mediated reaction in the peroxisomes is considered a minor metabolic pathway of alcohol metabolism.

The International Agency for Research on Cancer (IARC) has listed both ethanol and its major metabolite, acetaldehyde, as a carcinogen in humans [7]. The mechanisms underlying alcohol-induced cancer are still not well defined, although plausible events include: genotoxic effect of acetaldehyde, increased estrogen concentration, cellular stress, altered folate metabolism [8,9,10], and inflammation [11]. Amongst all, ethanol metabolism plays an important role in carcinogenesis. Ethanol is absorbed by the small intestine and later metabolized by alcohol dehydrogenases (ADH) into acetaldehyde in the liver [12]. When alcohol consumption is high, cytochrome P450 2E1 (CYP2E1) can also catalyze ethanol into acetaldehyde while producing reactive oxygen species (ROS) [9]. Emerging evidence indicates that the imbalance in ethanol metabolism may be markedly involved in alcohol-associated cancer (Figure 1).

Here, we review the organs involved in alcohol-related cancer, underlying potential molecular mechanisms, and current challenges as well as implications of in vivo experimental models used to study cancer pathogenesis in alcoholics. In addition, we describe our current limited understanding of the influence of alcohol on the host immune system and the development of possible effective immunotherapy for cancer patients with alcohol abuse.

## 2. Epidemiologic Evidence for Alcohol-Associated Cancers

Epidemiologic studies from the last decades unequivocally acknowledge chronic alcohol consumption as an important risk factor for the development of different types of cancers. Also, this effect is dose dependent. An international group of epidemiologists and alcohol researchers from the IARC concluded from the available epidemiological data that the occurrence of malignant tumors of the oral cavity, pharynx, larynx, esophagus, liver, colorectum, and female breast are causally related to the consumption of alcoholic beverages [9]. Many prospective and case-control studies show a two to three-fold increased risk for the esophageal cancer in people who consume 50 g of alcohol a day (equal to approximately a half bottle of wine), compared with non-drinkers [13,14,15,16].

A clear evidence of alcohol effects in upper aerodigestive tract (UADT) cancers is exhibited by significant increase in relative risks (RR) for cancer even at moderate daily doses of 25 g/day and with relative risks in four to six-fold range with higher rates of alcohol consumption [16]. Intake of more than 80 g alcohol per day leads to the RR ranging between 4.5 and 7.3 for hepatocellular carcinoma (HCC), compared with abstinence or consumption of less than 40 g per day [17]. The risk of HCC development shows a dose dependent relationship for the amount of ethanol consumed [16]. The incidence of HCC is predominantly associated with the condition of chronic inflammation that can lead to cirrhotic liver. Although rare, HCC in non-cirrhotic liver has also been reported and mostly occurs due to hepatitis B virus (HBV) infection [18], contamination with aflatoxin B1 [19], mutations of certain genes such as telomerase reverse transcriptase (*TERT*) and catenin beta 1 (*CTNNB1*), encoding β-catenin [20].

Numerous epidemiological studies have consistently demonstrated a dose-response relationship between chronic alcohol consumption and increase in the risk for breast cancer [21,22]. A meta-analysis of 38 epidemiological studies revealed that the risk of breast cancer for one, two, or three or more drinks per day increases by 10%, 20% and 40%, respectively [23]. These data indicate that nearly 4% of all newly diagnosed breast cancer cases in the US (approximately 8000 cases per year) occur due to alcohol abuse. Genetic epidemiology in breast cancer research describes subpopulation of women being more susceptible to alcohol-related breast cancer risk. In the Danish prospective cohort study, variant allele carriers of peroxisome proliferator-activated receptor gamma (*PPARG2*) Pro^12^Ala (rs1801282) polymorphism had a 20% increased risk of breast cancer per 10 g of alcohol consumed per day [24]. Epidemiological studies also demonstrate evidence for increased risk of estrogen receptor-positive (ER^+^) breast cancer due to alcohol intake [25].

Alcohol intake is considered to be associated with the increased risk for pancreatic cancer, but the degree of risk due to consumption pattern is still not clear. A meta-analysis of 32 studies revealed that high rates of drinking (≥3 drinks/day) were linked with a notable increase in RR (RR = 1.22) for pancreatic cancer [26]. A positive dose-response relationship between alcohol consumption and colorectal cancer was also reported in more than 50 prospective and case-control studies [27,28]. Collective data from eight cohort studies [28] and a meta-analysis [29] confirmed a RR of 1.4 for colorectal cancer in patients who consumed 50 g of alcohol per day compared with abstainers.

## 3. Alcohol: Tumor Initiator or Promoter?

Carcinogenesis initiation involves the interaction of the carcinogen with DNA, producing either a strand break or an adduct. Mutations caused by oxidative DNA damage include oxidized purines and pyrimidines, alkali labile sites and instability. Under certain circumstances errors in the altered- or initiated-cell could confer a selective growth advantage leading to tumor progression [30].

In vivo and in vitro studies from several groups provide insights into the effect of ethanol on initiation and progression of cancer. Previously, ethanol was considered a co-carcinogen, and not a carcinogen, since it failed to induce tumors in animals when given alone. This indicates that when alcohol is administered along with other cancer-inducing agents (i.e., carcinogens), it promotes cancer development. Most of the studies present alcohol as an “incomplete” carcinogen which cannot initiate mutagenesis but can enhance tumor growth in concert with small doses of other carcinogens. Several studies have reported that either simultaneous or alternative administration of ethanol with chemical carcinogen aggravates carcinogenesis, especially in UADT [31], mammary glands [32], liver [33] and large intestine [34] resulting in tumor promotion. However, multiple recent in vivo experiments in which mice and rats were fed with alcohol in their drinking water have identified ethanol as a direct carcinogen [21,22,35,36,37]. Several factors contribute to the alcohol mediated cancer initiation, including the actions of acetaldehyde, DNA methylation, induction of CYP2E1, and oxidative stress. The outcome of in vivo experiments on alcohol and cancer development depends to a large extent on the type of carcinogen, its dose, duration of exposure, and the route of alcohol administration. Local administration of alcohol to the oral or esophageal mucosa causes increase in the occurrence of tumors possibly due to its irritant effect [38].

In addition, it has also been demonstrated that short-term exposure to ethanol (12–48 h) increased migration and invasion in breast cancer cell lines and chronic ethanol exposure enhanced aggressiveness of the cancer [39]. Animal studies strongly support a critical role of alcohol exposure at a young age on breast cancer development. Experimental models have robustly demonstrated that dietary exposure to ethanol during puberty causes morphologic changes in mouse mammary glands, including increases in ductal branching and epithelial growth and breast density [40]. For other cancers of the digestive tract (e.g., stomach, pancreas, colon, and rectum), however, the results are controversial and remain elusive, possibly due to the differences in study design. Conclusively, alcohol appears to play a multifaceted role in the process of carcinogenesis. However, substantial data confirming its role as tumor initiator and/or tumor progressor in patients with different cancer is still not well explained and requires extensive investigation.

## 4. Alcohol and Different Types of Cancer

### 4.1. Liver Cancer

Alcohol consumption has been established as a leading cause of chronic liver diseases and HCC. It may lead to the development of HCC either through direct (genotoxic) or indirect mechanisms (development of cirrhosis) [6]. The relationship between alcohol and liver disease correlates with the amount of alcohol consumed over a lifetime and the overall dose-response relationship between alcohol consumption and the risk of liver cancer is linear [41]. The most probable mechanism underlying alcohol-related liver carcinogenicity is through development of liver cirrhosis, although other events such as hepatitis B infection [19], genetic mutations [21] or altered hepatic metabolism of carcinogens may also play a role [13]. Heavy alcohol intake in combination with chronic hepatitis C infection increases the risk for HCC twice as compared with the risk for hepatitis C alone. Furthermore, synergism between chronic alcohol abuse and hepatitis C infection are frequently related to hepatocarcinoma in Western countries [42,43]. Exome sequencing analysis of liver tumors identified association between specific risk factor, including alcohol consumption and mutational signatures [20]. Various gene alterations were reported in liver tumors from individuals with alcohol abuse.

### 4.2. Breast Cancer

More than 100 empirical studies have established a positive correlation between moderate or chronic ethanol consumption and the incidence of breast cancer in pre- and post-menopausal women [44]. It has been reported that alcohol intake of more than 27 drinks per week increases breast cancer risk in pre-menopausal women irrespective of the type of alcoholic beverage consumed [44]. Among post-menopausal women, consumption of more than six drinks per week increases breast cancer risk. The association between alcohol and breast cancer is attributed to the increased levels of estrogen in women consuming alcohol. Other plausible mechanisms include enhanced mammary gland susceptibility to carcinogenesis, increased mammary carcinogen DNA damage, and greater metastatic potential of breast cancer cells [6]. Multiple studies have documented an association between alcohol intake and increased risk of ER^+^ and/or estrogen receptor and progesterone receptor-positive (ER^+^/PR^+^) breast cancer [45,46,47,48]. The results of the meta-analysis comparing the highest versus the lowest alcohol consumption categories reported a 12% increase in the risk of ER^+^ tumors, a smaller positive association (7% increase in risk) with all ER^−^ tumors, and no association with ER^−^/PR^−^ or ER^−^/PR^+^ tumors [45]. One of the recent studies showed a strong correlation between alcohol and the risk of human epidermal growth factor receptor 2 (HER2)^−^ than HER2^+^ breast cancer [49]. Similarly, in another study, women consuming alcohol were diagnosed with luminal A (ER^+^, PR^+^, HER2^−^) breast cancers and women who refrain from drinking were diagnosed with luminal B breast cancers (ER^+^, PR^+^, HER2^+^) [50].

In vivo studies using mice and rats have shown that chronic alcohol consumption induces expression of pro-inflammatory cytokines and chemokines in white adipose tissue [51,52,53]. Recent studies from our laboratory has reported that chronic-binge alcohol induces adipose tissue inflammation in vivo in female mice [54]. Alcohol-induced chronic inflammation in breast adipose tissue creates microenvironment that is conducive to increased tumor cell proliferation, metastasis, and enhanced tumor-related angiogenesis. Increased oxidative stress and continuous secretion of pro-inflammatory cytokines by inflamed adipocytes can elicit epigenetic changes in pre-cancerous cells [55]. It is plausible that inflamed tissue microenvironment offers an ideal setting for tumor onset and progression, and alcohol acts as a major driving force.

### 4.3. Esophageal Cancer

Multiple prospective and case-control studies from different regions of the world showed a consistent association between the risk of squamous cell carcinoma and the consumption of alcoholic beverages. Regular consumption of about 50 g alcohol per day has been associated with a two-fold increase in the risk for esophageal cancer [6]. Chronic alcohol intake is frequently associated with secondary motility disorders and lower esophageal sphincter tone alteration [6]. These effects predispose to gastroesophageal reflux, esophagitis, and intestinal metaplasia. Findings on the association of alcohol and esophageal cancer have been argued. Some studies reported either no change or an increase in the risk of 1.5–4.0 times of adenocarcinoma of esophagus and gastric cardia with alcohol consumption [56], while others reported a decrease [57] in the association between alcohol intake and adenocarcinoma risk. The effect of alcohol and smoking on risk of esophageal cancers appears to be more additive and consistent.

### 4.4. Pancreatic Cancer

Findings obtained from multiple prospective cohort and case-control studies have illustrated an inconsistent association between alcohol consumption and pancreatic cancer risk. However, alcohol abuse has been known to be a major cause of chronic pancreatitis and a risk factor for type 2 diabetes mellitus, both of which are linked to pancreatic cancer [58]. An association between heavy alcohol consumption and pancreatic cancer among men that may be mediated by dose, duration, and pattern of alcohol intake, including binge drinking, was reported by Gupta et al. [59]. The biologic mechanism underlying an association between alcohol consumption and pancreatic cancer is not fully understood. However, a probable mechanism is through the development of chronic pancreatitis as a result of drinking alcohol (>80 g/day for 10–12 years) [60]. The functional relationship between inflammatory processes and cancer development is well established. Alcohol and its metabolites modulate metabolic pathways involved in the inflammatory response and carcinogenesis [58]. These diverse metabolic effects of alcohol can act collectively with other risk factors (dietary, environmental, and genetic factors) resulting in pancreatitis and diabetes mellitus and, ultimately, leading to the advancement of pancreatic cancer.

## 5. Potential Molecular Mechanisms

Ethanol is eliminated via its oxidation to acetaldehyde by ADH and acetaldehyde is subsequently converted to acetate by aldehyde dehydrogenase (ALDH). Various studies have indicated that a disproportion between the activities of ADH and ALDH play a crucial role in alcohol-induced neoplasms [61]. Significantly higher activity of different isoforms have been reported in esophageal cancer [62], liver cancer [63], and cervical cancer [64]. Disturbances between ADH and ALDH activities in cancer cells might be attributed to the polymorphism in the associated genes which has been dealt in detail in the following section.

**Figure 2:**
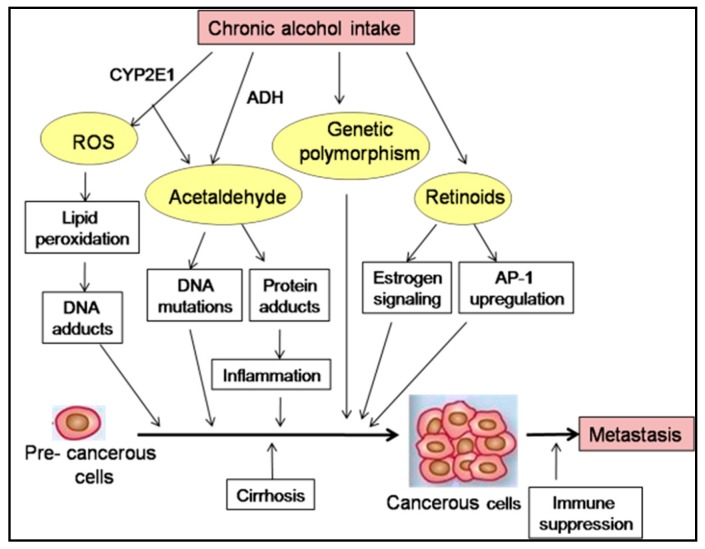
Various mechanisms by which alcohol may affect carcinogenesis. Ethanol is oxidized to acetaldehyde by ADH, which acts as a carcinogen and binds to DNA. This metabolism is modified by polymorphisms or mutations in the genes encoding metabolizing enzymes. Acetaldehyde can form hybrid-adducts with reactive residues (e.g., malondialdehyde adduct) acting on proteins, mediating lipid peroxidation and nucleic acid oxidation. Excessive alcohol consumption leads to the induction of CYP2E1 pathway and may indirectly contribute to acetaldehyde development and ROS production. Excessive alcohol enhances catabolism of retinoic acid by alcohol-induced CYP2E1. The interaction of retinoids with different signaling pathways, including estrogen signaling, may favor proliferation and malignant transformation of pre-cancerous cells. Chronic ethanol intake is also associated with the failure of immune surveillance of tumor cells.

The mechanisms of ethanol-induced carcinogenesis have been summarized in Figure 2. Some of the major and well studied mechanisms have been discussed in detail in this review.

### 5.1. Genetic Polymorphism

A multitude of studies suggest an association between genetic variants and alcohol consumption on cancer risk in humans. Here we summarize the published studies on the combined effects of alcohol drinking and polymorphisms in genes for ADH, ALDH, CYP2E1, and methylene-tetrahydrofolate reductase (MTHFR) on the risk of alcohol-related cancer.

The gene *ADH1B* shows several polymorphisms and has been associated with the risk of different cancers. Two studies in Asian populations found a significantly higher risk of cancer of upper aerodigestive tract, oral cavity, and hypopharynx in moderate or heavy drinkers carrying ADH1B*1/*1 genotype than those carrying ADH1B*1/*2 or ADH1B*2/*2 [65,66]. The enzyme encoded by ADH1B*1/*1 has only 1% and 0.5%, respectively, of the oxidation capacity of those encoded by ADH1B*1/*2 and ADH1B*2/*2. Another polymorphism in *ADH1C* modifies breast cancer risk. Premenopausal women carrying ADH1C*1 variant are found to be at a 1.8 times greater risk for breast cancer than women with other two genotypes [67]. In vitro studies reported that the enzyme subunits encoded by the ADH1C*1 allele metabolize alcohol to acetaldehyde two and half times faster than the ADH1C*2 allele [68]. The effect of *ADH1C* genotype and chronic alcohol consumption was noted on the risk of liver cancer in Caucasians [69]. Furthermore, a significantly higher risk of colorectal cancer has been reported in women who drank heavily and carried ADH1C*1/*1 genotype.

One of the recent meta-analysis study among Japanese population indicated Glu504Lys polymorphism of *ALDH2* gene as a candidate for susceptibility to esophageal cancer [70]. Several other studies show a significantly increased risk of esophageal cancer for Asian individuals who are heavy or moderate drinkers and carry ALDH2*1/*2 or ALDH2*2/*2 genotype compared to those who have an ALDH2*1/*1 genotype [65,71,72]. Several data lend support to the increased risk of liver cancer in alcoholics carrying genotypes ALDH2*1/*2 and ALDH2*1/*1 [73,74]. Individuals who are ALDH2*2 homozygous have null ALDH2 activity, and those who are heterozygous have about 6% residual activity leading to the increased accumulation of acetaldehyde.

Most of the studies revealed inconsistent findings on the effects of CYP2E1 polymorphism and alcohol consumption on various cancer risks. A meta-analysis of published reports showed that PstI/RsaI polymorphism of *CYP2E1* may increase the risk of HCC and, alcohol consumption increases the probability of developing HCC [75]. No risk associated with *CYP2E1* polymorphisms and esophageal squamous cell carcinoma with alcohol consumption was reported in Brazilian population [76], however, significant increased risk was noted for Asians with heavy alcohol intake who carry *CYP2E1c1/c1* or *CYP2E1c1/c2* genotypes, compared with non-drinkers [77]. It has been reported that *c2* variant allele show 10-times higher transcriptional activity, elevated protein levels, and increased enzyme activity compared to the *c1* allele [78].

Significant interactions between heavy drinking and MTHFR TT (homozygous variant) genotype have been reported for head and neck cancer (HNC) [79], esophageal cancer [80] and colorectal cancer [81]. Compared with individuals of CC (homozygous normal) genotype, those who carry TT or CT (heterozygous) genotype have approximately 30% and 65% MTHFR activity, respectively [82]. An increase in breast cancer risk has been reported among postmenopausal women who were homozygote TT for MTHFR C677T and were heavy drinkers, compared with non-drinkers who were homozygote CC [83]. Increased risk of HCC in patients with MTHFR *CC* genotype who consumed a high alcohol diet had been reported by Saffroy et al. [84].

### 5.2. Oxidative Stress

A mounting body of evidence suggest that cancer initiation and progression is closely linked to oxidative stress. The metabolism of ethanol leads to generation of ROS that serve as primary carcinogens due to their genotoxic effects on diverse cellular processes. ROS produced by CYP2E1 results in the accumulation of lipid peroxidation products such as malondialdehyde and 4-hydroxynonenal (4-HNE) which in turn forms exocyclic DNA adducts [85]. ROS can act as messengers in intracellular signaling pathways leading to the transformation of a normal cell to tumor cell [86]. These pathways alter cell cycle behavior by activating nuclear factor kappa-light-chain-enhancer of activated B cells (NF-κB) signaling pathway [87] and activator protein-1 (AP-1) (c-jun and c-fos) expression and promoting cell metastasis through the regulation of mitogen-activated protein kinase (MAPK) [86]. ROS accumulation leads to the upregulation of vascular endothelial growth factor (VEGF) and monocyte chemotactic protein-1 (MCP-1) [88], key mediators of tumor angiogenesis and metastasis. ROS mediated increase in the expression of metalloproteinases, MMP2 and MMP9, leads to breakdown of extracellular matrix, cell motility and thus favors tumor metastasis [89]. ROS mediated DNA damage and other effects of ROS are widely accepted as a cause for initiation and progression of breast cancer [90], HCC [88], lung cancer [91], and prostate cancer [92].

### 5.3. Retinoic Acid Metabolism

Retinoids (vitamin A and its derivatives) induce cellular growth, cellular differentiation, and apoptosis, thereby controlling carcinogenesis [93]. Lower hepatic vitamin A levels have been well documented in alcoholics. Excessive alcohol is known to hinder retinoid metabolism in multiple ways: (1) acts as a competitive inhibitor of vitamin A oxidation to retinoic acid; (2) enhances catabolism of retinoic acid by alcohol-induced CYP2E1; and (3) increases vitamin A mobilization from liver to extrahepatic tissues. Retinoid signaling is often compromised early in carcinogenesis, which suggests that a reduction in retinoid signaling may be required for tumor development. Retinoids exert a profound effect on other signaling pathways, including estrogen signaling in breast cancer [94]. Lower levels of serum retinol have been associated with higher risk of HNC among occasional drinkers by Chen et al. [95]. Previous experimental data supports that low hepatic retinoic acid (RA) levels in rats due to chronic alcohol administration may favor proliferation and malignant transformation of hepatocytes via upregulation of AP-1 (c-fos and c-jun) gene expression [96].

## 6. Animal Models

In order to extensively investigate the role of alcohol in the initiation and/or promotion of carcinogenesis and develop new potential therapies, there is a significantly growing interest to establish experimental models that could test the effect of alcohol exposure in vivo. Animal models have revolutionized our ability to investigate the molecular pathways and mechanisms underlying carcinogenesis induced by alcohol. Different rodent models are well known and have been used over the years to study cancer pathogenesis. The laboratory mouse is one of the best experimental models, due to the physiologic, genetic and molecular similarities to humans, its short lifespan, breeding capacity, and the limitless options offered by genetic engineering.

Here we provide a brief synopsis on three major types of animal models: (i) chemically induced models; (ii) genetically modified models; and (iii) xenograft models, and their relevance to alcohol research.

### 6.1. Chemically Induced Models

A wide range of chemical carcinogens has been proven capable of inducing cancers in experimental animals after prolonged or excessive exposures. However, these models do not completely resemble human pathogenesis. The genotoxic drug diethylnitrosamine (DEN) has been widely used to induce hepatic carcinoma in rodents [97], and is the most commonly used chemical to induce liver cancer in mice. The co-treatment of mice with DEN and CCl_4_ resulted in dramatic increase in the liver tumor incidence where 100% of the animals in the co-treatment group developed liver tumors [98]. Recent studies show that combination of DEN followed by alcohol exposure increase incidence of HCC promoted by underlying alcoholic liver disease [99]. Experimental model of chemically induced HCC in male BALB/c mice was developed in which DEN initiation with CCl_4_ and ethanol promotion induced a two-stage liver carcinogenesis mimicking the usual sequence of events observed in human HCC [100].

Carcinogenesis of mammary gland in rats is induced chemically using 7,12-dimethylbenz(a) anthracene (DMBA) or *N*-nitroso-*N*-methylurea (NMU) and has been utilized extensively to investigate hormone-dependent adenocarcinomas [101]. Use of *N*-nitrosobis(2-oxopropyl)amine (BOP) in the Syrian golden hamster [102] and 7,12-dimethylbenzanthracene (DMBA) in rats [103] are used for the development of pancreatic neoplasms. Transplacental induction of pancreatic ductal cancer by ethanol and 4-(methylnitrosamino)-1-(3-pyridyl)-1-butanone (NNK) in hamsters has been used to investigate a synergistic effect of alcohol drinking and cigarette smoking on fetuses [104]. In one of the study, DMBA-induced pancreatic carcinogenesis in mouse model verified the role of alcohol in inducing pancreatic adenocarcinoma [105]. Among the chemically induced colorectal cancer models, dimethylhydrazine (DMH) and its metabolites azoxymethane (AOM), and methylazoxymethanol (MAM) acetate are the widely used colon carcinogens [106]. Both AOM and DMH undergo metabolic activation by CYP2E1 to form reactive intermediates that elicit tumorigenesis. It has been reported that ethanol treatment in ACI/N rats significantly increased MAM-initiated large bowel tumorigenesis [107]. Chronic ethanol feeding in rats treated with DMH has been shown to significantly increase the number of aberrant crypt foci in colons [108].

### 6.2. Genetically Modified Models (GMM)

Genetically engineered mouse models mimic pathophysiological and molecular features of human carcinogenesis [109]. The classical gene targeting strategies entailed the disruption (knock-out) or substitution (knock-in) of an allele in embryonic stem (ES) cells. The knockout mouse models for enzymes metabolizing ethanol (ADH1, catalase and CYP2E1) [110,111], acetaldehyde (ALDH2, ALDH1A1 and ALDH1B1) [112,113,114] and enzymes involved in glutathione (GSH) synthesis (glutamate cysteine ligase catalytic subunit and glutamate cysteine ligase modifier subunit) [115,116,117] represent highly useful animal models for alcohol and carcinogenesis research. The common genetically engineered models of pancreatic cancer are based on *Kras* mutations and also include PDX-1-Cre/Lox-Stop-Lox (LSL)-Kras or p48/LSL-Kras mice, which have been modified by deletions or mutations of *Ink4*, *p53*, *Mist*, *Smad4* or *TGF-β* [118,119,120]. A broad range of genetically modified mice has been developed to investigate the pathophysiology of HCC. Transgenic mice over-expressing oncogenes (Myc protein, β-catenin), and growth factors (TGF-α, epidermal growth factor, fibroblast growth factor 19, platelet derived growth factor) represents good experimental models to investigate factors involved in malignant transformation of hepatocytes and its underlying mechanism [121,122].

During the last few years, more advanced techniques including genome editing, programmable nucleases, including zinc-finger nucleases (ZFNs) and transcription-activator-like effector nucleases (TALENs) have been developed to generate cancer models [123]. One study reported the use of TALEN-approach to edit the *β-catenin* gene in mouse liver to generate an efficient and physiologic liver cancer mouse model [124]. However, both ZFNs and TALENs are nuclease-based designs that are difficult to construct and have varying targeting efficiency. The recent advent of the clustered regularly-interspaced short palindromic repeats (CRISPR)-Cas9 system has revolutionized the field of cancer modeling. CRISPR is a powerful genome-editing tool that target specific genomic loci with a single-stranded guide RNA (sgRNA) [125]. The first study successfully utilizing CRISPR-Cas9 system to induce liver tumors was carried out by inoculating mice with sgRNAs targeting *Pten* and *p53* [126].

To date, numerous studies have demonstrated genetic modeling as a promising technique for developing tumor models. However, its potential to generate models for alcohol-induced cancers is yet to be explored.

### 6.3. Xenograft Models

The development of xenograft models has significantly improved cancer research and has been extensively used to study HCC. In xenograft models, the tumors are induced by injecting human cancer cells in the immune deficient mice, such as athymic or severe combined immune deficient (SCID) mice, either subcutaneously in the flank of mice, known as ectopic model or directly into the specific organ, known as orthotopic model. The first highly metastatic model of HCC (LCI-D20) in nude mice was developed by integrating HBV-DNA in the cellular DNA of LCI-D20 tumor cells [127]. An experimental mouse model to mimic the development of human alcohol-induced breast cancer was developed for the first time by subcutaneously injecting ER^+^ breast adenocarcinoma cells in the mammary gland of female immunocompetent C57BL/6 mice [128]. This study successfully demonstrated that even moderate alcohol consumption significantly stimulates breast tumor growth via induction of angiogenesis and VEGF expression. Different studies have used tumor engraftment in nude mice to study the response to chemotherapy treatment and suggest new potential treatment options for pancreatic cancer [129]. Utilization of these models should accelerate the generation of alcohol-induced tumors and develop targeted therapies to treat health issues associated with excessive alcohol consumption.

## 7. Alcohol-Induced Immune Modulation in Cancer

A large body of literature indicates that alcohol intake interferes with various aspects of innate and adaptive immune systems. Although many factors influence tumor growth and progression, evidence that highlights the role of host immune cells in controlling cancer growth and progression is accumulating. Once some cells are transformed into cancer cells, tumor immune surveillance, also known as tumor immunoediting, comes into play [130].

### 7.1. Innate Immune Surveillance

The innate immune response rapidly identifies cancerous and/or precancerous cells and destroys them. This response is recognized by inflammatory mediators (chemokines and cytokines) produced by an array of immune cells, such as natural killer (NK) cells, macrophages, neutrophils, and dendritic cells (DCs) [7]. NK cells actively recognize and prevent neoplastic development. Upon activation, NK cells produce cytokines and chemokines that generate inflammatory responses and activate adaptive immune response. Macrophages and neutrophils possess both anti-tumor activity as well as immune suppressive activity against tumor cells. DCs act as a connecting link between the innate and the adaptive wing of the immune system by identifying and presenting foreign molecules (i.e., antigens) to other immune cells.

Numerous reports have advocated a critical suppressive effect of alcohol on NK cell function. For example, alcohol consumption can inhibit the effector function of NK cells in the liver; suppress its cytolytic activity, block NK cell release from the bone marrow and significantly induce splenic NK cell apoptosis [131,132,133]. These findings have also been supported by some of the previous studies in mice where chronic alcohol administration inhibited NK cell activity [134], and reduced their number and lytic activity following a single binge equivalent of alcohol [135]. Another in vivo study in rats showed that acute alcohol exposure may cause ten-fold increase in lung metastases due to marked suppression of NK cell activity [136]. Decreased NK cytolytic activity has also been reported in human cancers, including HNC, breast, colorectal and prostate cancer [137,138].

The recruitment and infiltration of macrophages in the tumor microenvironment (also known as tumor-associated macrophages) activates them to support the malignant progression of cancer cells. Notably, multiple studies have revealed that prolonged alcohol exposure activate monocytes and macrophages, resulting in an increased production of pro-inflammatory cytokines, such as TNF-α, interleukins IL-1 and IL-6, and the chemokine IL-8 [139,140]. Chronic inflammation may further cause cellular transdifferentiation in organs, such as pancreas [141], stomach [142], intestine [143], and esophagus [144]. One of the recent study revealed that chronic alcohol intoxication exacerbates inflammation and triggers pancreatic acinar-to-ductal metaplasia, an initiating event leading to the development of pancreatic ductal adenocarcinoma [11].

An important requirement for effective immune responses against tumor is the presence of mature and functional DCs that recognize, process, and present tumor antigen. In HCC patients, the nature of DC abnormality, including defects in phenotypes, surface markers, endocytic capacity, and cytokine production has been clearly stated in various studies [145]. These DC have decreased expression of human leukocyte antigen–antigen D related (HLA-DR), lower levels of IL-2 and impaired endocytotic and allostimulatory capacity compared with DCs from normal controls. It is well established that alcohol attenuates the maturation of myeloid DCs as well as their antigen presenting ability and T cell stimulatory function [146,147,148,149]. Consequently, there is a reduced expression of HLA-DR, decreased IL-12 and increased IL-10 production [148]. Since alcohol causes maturation and functional defects in DCs, also apparent in HCC patients, correlating well with tumor development.

### 7.2. Adaptive Immune Surveillance

Several studies have illustrated the involvement of different T cell populations in controlling the tumor progression. A subtype of CD8^+^ T cells, expressing the memory phenotype (CD8^+^CD44^hi^) plays a crucial role in regulating metastasis [150,151,152]. A multivariate analysis of metastatic breast cancer patients established a correlation between increased CD3^+^CD4^+^ or CD8^+^CD28^+^ T cell subset and prolonged progression-free survival. On the contrary, increase in CD8^+^CD28^−^ T cells was linked with shortened progression-free survival [153]. In patients with gastric cancer, elevated peripheral blood levels of certain CD4^+^ T cell subpopulations, including Th22 (CD4^+^IL-22^+^IL-17^−^IFN-γ^−^) and Th17 cells were found to be associated with increased tumor progression [154].

A series of studies have been carried out in animals and cancer patients to delineate the effects of alcohol on T cell function. In one study, the role of ethanol and CD4^+^ T cells in controlling tumor growth was examined by implanting 201T human lung adenocarcinoma cell line into the lung of ethanol-fed BALB/c mice that also received anti-CD4 antibody [155]. These mice exhibited significantly larger tumors compared with non-ethanol fed control mice. In another study, alcohol consuming mice that were inoculated with B16BL6 melanoma showed marked reduction in the number of CD8^+^ T cells that specifically recognize a melanoma-specific antigen (i.e., gp100) compared with water-drinking control mice [156]. Also, in these ethanol-fed mice, CD8^+^CD44^hi^ T memory cells failed to expand. On the other hand, multiple studies in alcohol-consuming mice showed an increase in the percentage of cells known to suppress anti-tumor T cell immune responses, such as T regulatory cells (Tregs) (CD4^+^CD25^+^FOXP3^+^) and invariant natural killer T cells (iNKT) cells (CD3^+^NK1.1^+^) [156,157,158].

In contrast to the extensive studies involving T cells in anti-tumor immunity, the knowledge of B cells in anti-tumor immune responses are limited and controversial. B cells can recognize tumor antigens and produce anti-tumor antibodies. One of the studies reported that B cells enhanced T cell mediated anti-tumor immunity by producing anti-tumor antibody and presenting tumor-antigen to T cells [159]. They also showed that depletion of B cells enhanced B16 melanoma metastasis to the lung by inhibiting CD8^+^ T cell proliferation and Th1 cytokine production. On contrary, there are evidences suggesting that B-cell depletion could therapeutically enhance anti-tumor immune responses by decreasing IL-10 production from B cells [160]. In line with this report, another study showed that depletion of B cells exhibiting CD5^+^CD1d^hi^ IL-10^+^ phenotype inhibited tumor progression and enhanced anti-tumor immunity [161,162]. These investigators showed that chronic alcohol consumption impairs distribution and circulation of B cells in B6BL16 melanoma bearing mice by compromising B cell egress from the spleen [157]. Alcohol intake has been reported to hinder mature B cell circulation through modulation of the sphingosine-1-phosphate lyase-1 (SPL1) and sphingosine-1-phosphate receptor-1 (S1PR1) signaling pathway resulting in the impairment of T cell activation and anti-tumor cytokine production.

In summary, alcohol may modulate the immune system in a fashion that may favor tumor development and progression. Unravelling the details of immune alterations caused by alcohol exposure is crucial for developing more specific anti-tumor therapeutic strategies to ameliorate immune suppression in alcoholics.

## 8. Prospective Strategies for Tumor Immunotherapy in Alcoholics

Cancer immunotherapy that harnesses the host’s immune system has emerged as a promising method to control tumor progression and extend survival of cancer patients. Immunotherapies utilize diverse approaches, including stimulating effector mechanisms, and counteracting inhibitory and suppressive mechanisms against cancer. Strategies to activate effector immune cells include vaccination, adoptive cellular therapy, use of antibodies and administration of oncolytic viruses. Strategies to neutralize immune suppressor mechanisms include chemotherapy, use of antibodies to target immune checkpoint molecules and diminish Tregs function. However, there exists a large gap in knowledge as to how alcohol consumption affects anti-tumor immunity, and this severely hampers the development of effective immunotherapeutic approaches to treat cancer in people who have immune deficits due to chronic alcohol abuse.

Emerging research using animal models of alcohol-induced tumor indicates the development of promising strategies of cancer immunotherapy that could be successfully translated to humans. One such approach to recover anti-tumor immunity by targeting iNKT cells and CD8^+^ T cells. Previously, the B16BL6 melanoma model established an association between alcohol intake and increased iNKT cell number [156,157,158]. With increasing tumor growth, the crosstalk between alcohol and tumor cells leads to iNKT cell anergy. Therefore, development of immunotherapeutic strategies inhibiting iNKT cell anergy through blocking the interaction between alcohol and tumor cells could emerge as a plausible therapeutic outcome in alcoholics. Another reasonable approach is the blockade of programmed cell death protein 1/programmed death-ligand 1(PD-1/PD-L1) and natural killer group protein 2A (NKG2A) signaling pathway using antibodies or small interfering RNA (siRNA) to prevent iNKT cell anergy induced by glycolipid antigen, α-galactosylceramide, and enhance anti-tumor activity in alcohol-induced tumor bearing mice [163,164]. Myeloid-derived suppressor cells (MDSC) and iNKT cells are key inhibitory cells that modulate CD8^+^ T cell function in mouse model of alcohol-induced tumors. Targeting these cells may lead to the restoration of CD8^+^ T cell function. Also, in these mice, immunotherapy targeting IL-15/IL-15Rα could be another strategy to boost CD8^+^ T cell function [164]. Targeting underlying molecular basis of interaction between alcohol and cancer cells, leading to the modulation of sphingosine-1-phosphate/receptor 1 (S1P/S1PR1) signaling pathway and impairment of mature B cell circulation could be another promising approach. With continued research and validation of a combination of different strategies may produce more efficient therapeutic regimen for alcoholics with cancer.

## 9. Conclusions and Future Approaches

Emerging scientific and clinical evidences support a causal association of alcohol consumption and cancers of different organs. Considering the increasing trends in adolescent drinking and binge drinking among young people, further studies are needed on drinking patterns, dose-response, alcohol consumption during specific periods of life, and genetic differences in the development of cancer. The mechanisms underlying ethanol-mediated carcinogenesis and tumor progression are very intricate and it is likely that a combination of contributing factors work synergistically. Evidence shows an indiscriminate role of host immune cells in controlling cancer growth and development. However, substantial data describing the specific interaction between alcohol and immune response in cancer patients or experimental animal models are still lacking. Undoubtedly, more mechanistic research is required to unravel the intricate association between alcohol and cancer. A broad range of animal models has been developed to define cancer pathogenesis and to test novel drug candidates. The experimental models have proven to be a useful tool in understanding cancer pathologies in alcoholics. Utilization of advanced animal models should pave the way for the development of targeted therapies to treat cancer in patients with alcohol abuse.
